# Gambling and problem gambling among male junior elite footballers in Sweden: a cross-sectional study of prevalence, age differences and correlates

**DOI:** 10.1136/bmjsem-2026-003241

**Published:** 2026-05-14

**Authors:** Anders Nilsson, Håkan Wall, Johanna Gripenberg, Tobias H Elgan

**Affiliations:** 1 Clinical neuroscience, Karolinska Institutet, Stockholm, Sweden; 2 Stockholm Prevents Alcohol and Drug Problems (STAD), Centre for Psychiatry Research, Region Stockholm, Stockholm, Sweden

**Keywords:** Soccer, Football, Psychiatry, Psychology, Prevention

## Abstract

**ABSTRACT:**

**Objective:**

To examine gambling behaviours and problem gambling among Swedish male junior elite football players, including differences between players below and above the legal gambling age, and to identify gambling-related risk factors.

**Methods:**

A cross-sectional survey was conducted among players in the U16, U17 and U19 teams of the 32 clubs in the two highest divisions. In total, 741 players responded (43.3%). Past-year problem gambling was assessed using the Problem Gambling Severity Index and depressive symptoms using the Patient Health Questionnaire-9. Multivariable logistic regression was used to examine correlates of problem gambling.

**Results:**

Overall, 32% had gambled in the past year, with higher participation among players who had reached the legal gambling age (63.2% vs 22.4%). Problem gambling was nearly two times as common in this group (13.4%) compared with younger players (7.5%). Slots participation showed the strongest association with problem gambling followed by depressive symptoms. Awareness of gambling policies within clubs was low (15%).

**Conclusions:**

Gambling and problem gambling are more common among junior elite footballers, including underage players, than in the general population. The sharp increase in gambling after reaching the legal age, combined with low policy awareness, highlights the need for preventive interventions, clearer gambling policies and education within football clubs.

**WHAT IS ALREADY KNOWN ON THIS TOPIC**

- Elite athletes, including footballers, are at higher risk for gambling problems compared with the general population.
- High-speed gambling, such as slots and in-play betting, is associated with problem gambling due to its addictive nature.
- Gambling has become increasingly normalised in football through sponsorships and advertising from gambling companies, contributing to elevated gambling involvement among players.

**WHAT THIS STUDY ADDS**

- Problem gambling is common among male Swedish junior elite footballers, including among those under the legal gambling age; 7.5% of players under 18 and 13.4% among those 18–19 years old met criteria for problem gambling.
- Participation in slots emerged as the strongest predictor of problem gambling, while sports betting was the most common gambling type overall.
- Gambling activity increased sharply after players reached the legal gambling age of 18.
- Player awareness of gambling-related policies within their clubs was low.

**HOW THIS STUDY MIGHT AFFECT RESEARCH, PRACTICE OR POLICY**

- There is a need for preventive intervention strategies within football clubs, including education for junior players, early preventive messaging and decreased gambling availability for those under 18, and systematic policy implementation.
- Results can inform discussions about policy changes, advocating for reduced gambling sponsorships and strengthening regulations of athlete gambling to protect young players and the sport’s integrity.
- The study underscores the importance of developing gambling policies within clubs.
- Future research could explore the co-creation and implementation of preventive interventions in football clubs and assess their effectiveness.

## Background

 Gambling is a widespread activity that can be associated with significant harm, including financial distress, mental health problems and addiction.^1^ Gambling has become increasingly digitised,^2^ making it more accessible through online platforms and mobile apps. This trend raises concerns in sports contexts, where gambling is often normalised through sponsorships and advertising.^3^ For example, during the 2024–2025 season, nearly half of Europe’s top football leagues had a gambling operator as ‘title sponsor’ and 296 of 442 teams in the European top flights had signed with at least one gambling operator.^4^ Many clubs are also sponsored by companies providing gambling-like products such as cryptocurrency trading.^5^ Football, as the most popular sport globally, is closely intertwined with sports betting, and research suggests that athletes are an at-risk group for gambling problems due to traits such as competitiveness, sensation-seeking and risk-taking.^6^

Previous studies indicate that elite adult football players exhibit higher rates of problem gambling compared with the general population.^7 8^ For example, research among Swedish male elite footballers found that 60% had gambled in the past year, and 6.6% met criteria for problem gambling, with high-speed games such as slots and bingo being strong predictors of gambling problems.^8^ While sports betting was the most common gambling activity, it was not associated with problem gambling, highlighting the complexity of gambling behaviours.

Despite growing evidence that gambling behaviours can begin early and that exposure to gambling advertising during adolescence increases the risk of future gambling problems, little is known about gambling among junior elite football players. This knowledge gap is concerning, given that the legal age for gambling in Sweden and most European countries is 18 years, yet anecdotal reports and preliminary data suggest that underage gambling is common.^9^,^11^ Adolescents may be particularly vulnerable due to developmental factors such as impulsivity and limited risk perception, combined with peer norms and the pervasive presence of gambling in football culture.^12^ Junior elite football players thus represent a unique risk group, combining developmental vulnerability with intensive exposure to gambling through the football environment.

The current exploratory study aims to address this gap by examining gambling behaviours and problem gambling among male junior elite football players in Sweden, including those under 18 years of age. Specific research questions pertain to the prevalence of gambling and problem gambling among junior elite footballers, differences depending on whether players have reached the legal gambling age, predictors for problem gambling and awareness of gambling policies. Understanding gambling patterns among junior players is critical for developing effective prevention efforts within football clubs and federations. Such knowledge can inform policy discussions on gambling sponsorships, education initiatives and early intervention measures aimed at reducing gambling-related harm among young footballers.^13^

## Methods

### Design

A cross-sectional design including junior players from the 32 clubs in Superettan and Allsvenskan, Sweden’s two highest football leagues. The study was part of a larger research project; results concerning senior players have been reported previously.^8 13^

### Participants

The participants were registered in the three oldest junior teams (boys aged up to 16/17/19 years) from the 32 top football clubs in Sweden. The participants could be considered elite athletes, competing at the highest national level possible at their age group and were part of professional club organisations.^14^

### Procedure

In collaboration with the Swedish Professional Football Leagues, email contact lists for junior players from 89 of the 95 eligible teams were obtained in February 2021. All players (N=1713) were invited via email to complete an online survey. Non-respondents received up to five reminders. Data were collected between February and November 2021. Participants provided informed consent before responding.

### Measures

The survey included background questions and scales assessing problem gambling and mental health. The 9-item Problem Gambling Severity Index (PGSI)^15^ measured past-year gambling (score range 0–27); a cut-off of ≥3 indicated problem gambling. Participants also reported engagement in eight gambling types (involving money) during the past 12 months (eg, physical or online sports betting, poker, casino games, slots). The 9-item Patient Health Questionnaire (PHQ-9)^16^ assessed depressive symptoms over the past 2 weeks (score range 0–27; cut-off ≥5 indicated elevated depressive symptoms). A question on whether they were aware of a written gambling policy in their club was included.

### Data preparation

To compare participants under and over 18 years of age, we created a variable called under 18. Age was missing for 125 participants; among these, 71 who reported playing in U16 or U17 teams were classified as under 18, as eligibility for these teams requires being younger than 18. Participants in U19 teams (n=46) who did not report age were excluded from age-based comparisons because underage players can also compete in U19; 35% of U19 players who reported age were under 18.

### Data analyses

We conducted a multivariable logistic regression to examine factors associated with problem gambling (PGSI≥3). We had 62 events of problem gambling, which allowed us to have 5–6 predictor variables. To keep the model parsimonious and avoid overfitting, we used five predictor variables, including past year participation in slots, betting, casino games and poker, as well as depressive symptoms (PHQ-9≥5). These predictors are well-known risk factors for problem gambling.^8 17 18^ Model performance was assessed using the area under the receiver operating characteristic curve, Brier score, Hosmer-Lemeshow goodness-of-fit test and variance inflation factors to check multicollinearity. Age group was not included in the model since it degraded the model’s performance.

In addition, univariate analyses were conducted to compare gambling behaviours between participants under 18 years of age and those aged 18 years or older (18+). Differences in prevalence for binary variables were assessed using Pearson’s χ^2^ tests; when expected cell counts were below five, Fisher’s exact test was used. Pearson’s χ^2^ tests were conducted without continuity correction. Differences in the number of gambling formats were examined using Welch’s two-sample t-test, which does not assume equal variances. Percentages and descriptive statistics were calculated using available data for each variable, and sample sizes therefore vary slightly due to item-level missingness. All analyses were conducted using R V.4.3.1.

## Results

The sample included 741 participants, which yielded an overall response rate of 43.3%. Response rates varied substantially between clubs, ranging from 3.1% to 85.9%, and 11 of the 32 clubs had response rates exceeding the overall response rate. See table 1 for background characteristics of the participants.

**Table 1:** Background characteristics, N=741

Variable	N=741
Age, mean (SD)	16.7 (1.7)
Education, n (%)	
Not completed compulsory school	125 (16.9)
Compulsory school	357 (48.3)
Secondary school	253 (34.2)
University, higher education	–
Other tertiary education	4 (0.6)
Changed place of residence due to football, n (%)	94 (12.7)
Accommodation through the club, n (%)	46 (6.3)
Training hours per week, median (IQR)	10 (4)
Clubs last 5 years, median (IQR)	2 (0)
Changed clubs past 12 months, n (%)	164 (22.2)

### Problem gambling and gambling participation

A total of 32% gambled last year. Among adult junior players (18+) this figure was 63.2% and 22.4% for the under 18 group. Problem gambling (PGSI≥3) was observed in 9.4% of the total sample and was almost two times as common among 18+ players compared with those under 18 years (table 2). Moreover, 18+ players reported significantly higher gambling involvement than those under 18; the largest differences were for sports betting and casino games. 18+ players also engaged in about four times more gambling formats on average compared with under 18. A total of 15.0% of all players were aware of a written gambling policy within their club.

**Table 2:** Comparison of gambling behaviours by age group

Variable	Under 18 (n=532–541)	18+ (n=142–144)	Test	Statistic	P value
PGSI≥3	7.5%	13.4%	Pearson χ²	4.82	0.028
Sports betting	11.7%	51.7%	Pearson χ²	112.69	<0.001
Bingo	2.6%	6.3%	Fisher’s exact	–	0.038
Casino games	2.0%	20.8%	Pearson χ²	71.28	<0.001
Horse betting	0.9%	5.6%	Fisher’s exact	–	0.002
Lotteries	10.8%	20.3%	Pearson χ²	9.20	0.002
Number games	1.7%	2.8%	Fisher’s exact	–	0.488
Poker	5.4%	24.5%	Pearson χ²	48.33	<0.001
Slots	1.7%	13.2%	Pearson χ²	38.48	<0.001
Number of gambling formats, mean (SD)	0.36 (0.83)	1.44 (1.72)	T-test	−7.34	<0.001

Percentages are calculated based on non-missing observations within each age group. Sample sizes therefore vary slightly across variables owing to item-level missingness (under 18: n=532–541; 18+: n=142–144). Pearson’s χ² tests were used without continuity correction when expected cell counts were ≥5; Fisher’s exact tests were applied otherwise. Differences in the number of gambling formats were assessed using Welch’s two-sample t-test.

PGSI, Problem Gambling Severity Index.

### Regression model results

In a multivariable logistic regression predicting problem gambling (PGSI≥3) among all junior players regardless of age, slots participation and depression measured with PHQ-9≥5 were significantly associated with higher odds, adjusting for sports betting, casino games and poker (table 3). Sports betting and poker showed borderline associations.

**Table 3:** Results from multivariable logistic regression with Problem Gambling Severity Index three or more points as outcome variable

Variable	OR (95% CI)	P value
PHQ-9≥5	2.06 (1.14 to 3.66)	0.015
Sports betting	1.76 (0.89 to 3.37)	0.093
Casino games	1.37 (0.47 to 3.67)	0.548
Poker	2.08 (0.84 to 4.83)	0.098
Slots	3.34 (1.14 to 9.63)	0.026

The model demonstrated moderate discrimination (area under the receiver operating characteristic curve =0.721), acceptable calibration (Hosmer-Lemeshow p=0.426) and a Brier score of 0.073, outperforming the null model by ~6.6% on Brier skill. Multicollinearity was low (all variance inflation factors <2).

PHQ-9, 9-item Patient Health Questionnaire.

## Discussion

This study examined gambling behaviours and problem gambling among male junior elite footballers in Sweden. Gambling was common, with 63.2% of players aged ≥18 and 22.4% of underage players reporting past-year gambling despite legal restrictions. Problem gambling (9.4%) was substantially more common compared with the general population, where 2.2% (CI 1.9 to 2.5) have been found to have problem gambling,^7^ and somewhat elevated in males of the same age group from the general population.^18^ Online sports betting was the most common form of gambling, whereas online slots were most strongly associated with problem gambling, with more than a threefold increase in the odds of having PG. This confirms previous studies that identify online slots as being particularly associated with problem gambling among young males.^14^ Furthermore, symptoms of depression were also significantly associated with increased risk of problem gambling, consistent with previous studies.^13^

The prevalence of problem gambling observed in our study is comparable with previous studies on junior elite athletes,^19^ although differences in methods impede direct comparisons. Consistent with earlier research,^15^ our study shows that many underage players gamble. This demonstrates that gambling is available earlier than stipulated by regulation. It is possible that minors may gain access to online gambling through older teammates or friends or through gambling operators without a Swedish licence, although this warrants further study.

Previous studies have highlighted the importance of peer norms in shaping gambling behaviour,^20^ in particular within sports clubs’ settings.^21 22^ This could be seen as part of the ‘gamblification’ of sports, where sports betting is normalised, with a heightened risk of harmful gambling among sports participants.^23^ Gambling is often seen as a natural extension of sports,^24^ and a part of peer rivalry and competition among young males.^25^ It is likely that some of the gambling among junior elite athletes could be explained by these processes, alongside attitudes and behaviours among coaches and other family members.^11 19^

In this cohort, gambling is a particularly relevant target for prevention efforts. Yet only 15% of participants were aware of any club policy regarding gambling. This points to a policy vacuum, where gambling sponsorship is central to the economy of the clubs, while their players are at risk of experiencing severe harm from gambling. Furthermore, players under the age of 18 are legally prohibited from gambling but are exposed to gambling through older teammates and club sponsorships. Problem gambling is associated with significant harm for the individual, including detrimental effects on psychological and physical health.^1^ There is reason to believe that problem gambling has a negative impact on sports performance,^26 27^ and it should be in the interest of the football clubs to implement systematic preventive measures, including education and policies restricting gambling among young players.

### Limitations

This study has several limitations. It has a cross-sectional design which precludes conclusions about causality between gambling behaviour and mental health outcomes. Data were self-reported, which may involve recall and social desirability bias. The sample consisted of male footballers from Sweden’s top two leagues, limiting the generalisability of the findings to female players and other competitive levels. Although all players were invited to respond, the resulting sample size limited the ability to adjust for a wider range of potential confounders. The substantial variability in response rates between clubs may also have affected the representativeness of the sample, and selection and participation bias may be present if clubs or individuals with differing levels of gambling involvement were more or less likely to participate. Finally, data were collected during the COVID-19 pandemic, which influenced football activities and gambling patterns. Strengths are that the study comprises a large sample of footballers from Sweden’s top leagues and that validated measures were used to assess problem gambling and mental health.
